# An appropriate ammonium: nitrate ratio promotes the growth of centipedegrass: insight from physiological and micromorphological analyses

**DOI:** 10.3389/fpls.2023.1324820

**Published:** 2023-12-19

**Authors:** Dong-Li Hao, Jin-Yan Zhou, Ling Li, Jia Qu, Xiao-Hui Li, Rong-Rong Chen, Wei-Yi Kong, Dan-Dan Li, Jian-Jian Li, Hai-Lin Guo, Jian-Xiu Liu, Jun-Qin Zong, Jing-Bo Chen

**Affiliations:** ^1^ The National Forestry and Grassland Administration Engineering Research Center for Germplasm Innovation and Utilization of Warm-Season Turfgrasses, Jiangsu Key Laboratory for the Research and Utilization of Plant Resources, Institute of Botany, Jiangsu Province and Chinese Academy of Sciences (Nanjing Botanical Garden Mem. Sun Yat-Sen), Nanjing, China; ^2^ Department of Agronomy and Horticulture, Jiangsu Vocational College of Agriculture and Forest, Jurong, China; ^3^ Sanya Nanfan Research Institute of Hainan University, Sanya, China

**Keywords:** NH4 +:NO3 -ratio, stomatal aperture, root and stem structure, nitrogen use efficiency, carbon sequestration, centipedegrass

## Abstract

Reasonable nitrogen fertilizer application is an important strategy to maintain optimal growth of grasslands, thereby enabling them to better fulfil their ecological functions while reducing environmental pollution caused by high nitrogen fertilizer production and application. Optimizing the ammonium (NH_4_^+^):nitrate (NO_3_^-^) ratio is a common approach for growth promotion in crops and vegetables, but research on this topic in grass plants has not received sufficient attention. Centipedegrass, which is widely used in landscaping and ecological protection, was used as the experimental material. Different NH_4_^+^:NO_3_^-^ ratios (0: 100, 25:75, 50:50, 75:25, 100:0) were used as the experimental treatments under hydroponic conditions. By monitoring the physiological and morphological changes under each treatment, the appropriate NH_4_^+^:NO_3_^-^ ratio for growth and its underlying mechanism were determined. As the proportion of ammonium increased, the growth showed a “bell-shaped” response, with the maximum biomass and total carbon and nitrogen accumulation achieved with the NH_4_^+^:NO_3_^-^ ratio of 50:50 treatment. Compared with the situation where nitrate was supplied alone, increasing the ammonium proportion increased the whole plant biomass by 93.2%, 139.7%, 59.0%, and 30.5%, the whole plant nitrogen accumulation by 44.9%, 94.6%, 32.8%, and 54.8%, and the whole plant carbon accumulation by 90.4%, 139.9%, 58.7%, and 26.6% in order. As a gateway for nitrogen input, the roots treated with an NH_4_^+^:NO_3_^-^ ratio of 50:50 exhibited the highest ammonium and nitrate uptake rate, which may be related to the maximum total root length, root surface area, average root diameter, root volume, and largest root xylem vessel. As a gateway for carbon input, leaves treated with an NH_4_^+^:NO_3_^-^ ratio of 50:50 exhibited the highest stomatal aperture, stomatal conductance, photosynthetic rate, transpiration rate, and photosynthetic products. The NH_4_^+^:NO_3_^-^ ratio of 50:50 treatment had the largest stem xylem vessel area. This structure and force caused by transpiration may synergistically facilitate root-to-shoot nutrient translocation. Notably, the change in stomatal opening occurred in the early stage (4 hours) of the NH_4_^+^:NO_3_^-^ ratio treatments, indicating that stomates are structures that are involved in the response to changes in the root NH_4_^+^:NO_3_^-^ ratio. In summary, we recommend 50:50 as the appropriate NH_4_^+^:NO_3_^-^ ratio for the growth of centipedegrass, which not only improves the nitrogen use efficiency but also enhances the carbon sequestration capacity.

## 1 Introduction

Grasslands are known as barriers for ecological protection, contributors to soil formation, atmospheric filters, green granaries, and climate regulators. Globally, grasslands store 306-330 Pg C and are the main terrestrial carbon storage system, accounting for one-third of total terrestrial carbon storage (Tang et al., 2018). Maintaining the fine growth of grasslands is a precondition for them to fulfil their ecological functions (Smith, 2014). The extensive application of nitrogen fertilizer is the most important nutrient guarantee for the high yield and quality of grasslands (Li et al., 2022). However, 1) 70% of China’s nitrogen fertilizer production is dependent on coal rather than natural gas, thereby producing much more CO_2_ than developed countries. 2) Nitrogen use efficiency in China is 30%-40% (Hao et al., 2023), and the remaining portion either enters groundwater leading to pollution or escapes into the air in the form of N_2_O, causing ozone layer destruction and exacerbating global warming (Ravishankara et al., 2009). N_2_O can exist in the atmosphere for a long time (>120 years), and its greenhouse effect is 265-300 times that of equivalent CO_2_. Nitrogen fertilizer application is the main source of N_2_O emissions, which accounts for 44% of atmospheric N_2_O emissions caused by human activity and 77% of N_2_O emissions caused by soil (Akiyama et al., 2009). Grasslands account for 17-30% of the total natural soil emissions (Xu et al., 2018). Previous studies have shown that an average increase of 5% to 15% in nitrogen use efficiency can reduce N_2_O emissions by 30% (Akiyama et al., 2009). Therefore, the key to controlling grassland N_2_O emissions is to improve the nitrogen use efficiency of grass plants. 3) Excessive application of nitrogen fertilizer leads to a sharp decrease in soil organic matter and a serious decline in soil carbon storage and control (Khan et al., 2007), which restricts the soil carbon pool, the largest carbon pool in the biosphere, from playing a role in stabilizing carbon and fixing carbon. 4) Grasslands can intercept lost nitrogen fertilizer in farmland soil and alleviate a series of environmental problems, such as water eutrophication and atmospheric active nitrogen pollution, caused by low nitrogen fertilizer utilization efficiency in farmland (Shaddox et al., 2016). 5) The improvement of nitrogen use efficiency in grass plants would strengthen the ecological functions of grasslands, such as carbon sequestration and carbon sinks. Improving the nitrogen use efficiency of grass plants is a necessary strategy to achieve a double-win of ensuring stable growth of grassland and reducing the negative environmental effects caused by high nitrogen fertilizer production and application.

Ammonium and nitrate are the two main forms of inorganic nitrogen in soil. Many studies have shown that under the same applied concentration, the appropriate ammonium (NH_4_^+^): nitrate (NO_3_^-^) ratio promotes plant growth and improves nitrogen use efficiency. Notably, those studies mainly concentrate on Chinese cabbage (Chen et al., 2005; Hu et al., 2017), strawberry (Tabatabaei et al., 2006), cabbage (Zhang et al., 2007), tomato (Liu et al., 2017), maize (Wang et al., 2019b), coffee (Carr et al., 2020), purple coneflower (Ahmadi et al., 2021a; Ahmadi et al., 2021b; Ahmadi et al., 2022), pecan (Chen et al., 2021), soybean (Raza et al., 2021), Chinese kale (Wang et al., 2021), wheat (Yang et al., 2021), blueberry (Zhang et al., 2021), flowering Chinese cabbage (Zhu et al., 2021), lettuce (Du et al., 2022), and blackberry (Wei et al., 2023), with little attention given to grass plants. The growth promotion triggered by an appropriate NH_4_^+^:NO_3_^-^ ratio is attributed to the accumulation of more carbon and nitrogen (Chen et al., 2005), a higher leaf area and photosynthetic rate (Tabatabaei et al., 2006; Hu et al., 2017; Liu et al., 2017; Wang et al., 2019b; Carr et al., 2020; Raza et al., 2021; Zhang et al., 2021), a higher chlorophyll concentration (Liu et al., 2017; Raza et al., 2021), increased auxin synthesis (Wang et al., 2019b), improved absorption of H_2_PO_4_^–^, K^+^, Ca^2+^, and Mg^2+^ nutrients and a suitable proportion of nitrogen assimilation and storage (Zhang et al., 2007; Carr et al., 2020), improved root growth and root/shoot ratio (Ahmadi et al., 2021b; Raza et al., 2021; Zhang et al., 2021), or maintenance of the pH value and NH_4_^+^:NO_3_^-^ ratios of the nutrient solution (Wang et al., 2021; Zhu et al., 2021). However, information regarding the role of plant micromorphological structure in growth promotion and the early-stage response of the above physiological parameters to the changes of different NH_4_^+^:NO_3_^-^ ratios is still lacking. Based on the above background knowledge, centipedegrass, which is used as a typical landscape and ecological restoration grass, was used in this study as the experimental material. By monitoring the physiological and morphological changes under different NH_4_^+^:NO_3_^-^ ratio treatments, the appropriate NH_4_^+^:NO_3_^-^ ratio for growth and its underlying mechanism were determined. The recommended NH_4_^+^:NO_3_^-^ ratio would facilitate the growth of grass, enabling it to better function in ecological protection. This practice would simultaneously alleviate a series of environmental problems caused by low nitrogen use efficiency.

## 2 Materials and methods

### 2.1 Plant growth condition

Stolons of the centipedegrass were collected from field plots in the turfgrass nursery in Nanjing Botanical Garden Mem. Sun Yat-Sen, China. The stolon with the top three nodes was obtained by cutting off from these stolons and cultured in water for 7 days to allow root emergence (Xu et al., 2023). Then, uniform seedlings were subjected to different NH_4_^+^:NO_3_^-^ ratio treatments under hydroponic conditions. The nutrient solutions were composed of 0.5 mM nitrogen (with different NH_4_^+^:NO_3_^-^ ratios), 0.3 mM KH_2_PO_4_, 0.35 mM K_2_SO_4_, 1 mM CaCl_2_, 1 mM MgSO_4_.7H_2_O, 20 μM EDTA-Fe, 20 μM H_3_BO_3_, 9 μM MnCl_2_.4H_2_O, 0.77 μM ZnSO_4_.7H_2_O, 0.32 μM CuSO_4_.5H_2_O, and 0.39 μM Na_2_MoO_4_.2H_2_O. The NH_4_^+^:NO_3_^-^ ratio treatments were 0:100, 25:75, 50:50, 75:25, and 100:0. Each treatment contained 3 replicates. Each replicate included 12 seedlings planted in 3 L of nutrient solution. The pH of the nutrient solution was 5.5. The nutrient solution was renewed every three days. The room temperature was 28 °C, the relative humidity was 70%, the photosynthetic photon flux density was 500 μmol·m^−2^·s^−1^, and the photoperiod was 12 h/12 h (day/night). The seedlings were harvested after one month of treatment.

### 2.2 Growth parameter determination

The root parameters were obtained through a root scanner. Briefly, the root system was cut from the plant and then put into the sample plate without blocking each other. Four parameters, including total root length, root surface area, root volume, and average root diameter, were obtained by a root scanner. The plant height and internode length were measured with a ruler. The stem diameters were measured using a Vernier calliper. The number of nodes was obtained by counting.

### 2.3 Histochemical staining

The roots in the mature zone (5 cm from the root tip) and the 5^th^ stems of centipedegrass that received one month of treatment were used for the staining test. The experimental procedure is referred to in the following literature (Wang et al., 2019c). First, small pieces of the roots and stems were immersed in a solution containing phosphate buffer (pH 7.2) and 2.5% glutaraldehyde (4°C, overnight). Following dehydration with ethanol and infiltration with epoxy propane, the samples were soaked and embedded in paraffin. A 1 μm sample was obtained by using a Leica ultramicrotome. After deparaffinization with xylene and washing with ethanol, the samples were stained in 1% safranin-O for 2 h and dehydrated with different concentration gradients of ethanol (50%, 70%, and 80%) for 5 s, respectively. Then, the samples received 15 s of 0.5% fast green staining. Following washing with 95% ethanol and 100% ethanol, the sections were mounted using neutral balsam. Safranin-O stained the xylem vessel red; fast green stained the phloem sieve tube green. The structures of the samples were photographed using the Mshot Image Analysis system under a light microscope at 5x and 40x magnification. The number of vascular bundles was directly counted, and the area of the large xylem vessels was measured by the Mshot Image Analysis system.

### 2.4 Stomatal aperture and photosynthetic parameters

The second leaves from the top of the centipedegrass were taken and cultivated under different NH_4_^+^:NO_3_^-^ ratio treatments. Then, the leaves were placed in 0.5% KCl (pH 5.8) for 1.5 h. The solution clinging to the leaves was then carefully wiped off, nail polish was applied on the leaf surface, and temporary slides were created when the nail polish was dry. Photos were taken using the Mshot Image Analysis system under 40x magnification, and measurements of the length and width of the stomata were conducted by ImageJ software. The stomatal aperture was obtained by width/length. Each treatment contained data from at least 45 stomata.

Gas-exchange measurements were conducted in the totally expanded top second leaves of plants receiving different nutrient treatments using a Li-COR 6800 portable photosynthesis system. The rate of CO_2_ assimilation (Pn), transpiration rate (Tr), and stomatal conductance (gs) were determined under light-saturated conditions with a photosynthetic photon flux density (PPFD) of 1200 μmol m^−2^ s^−1^ at 25°C and with a reference CO_2_ concentration of 400 ppm.

### 2.5 Carbon and nitrogen content

After one month of treatment, the roots were soaked in a 0.1 mM CaSO_4_ solution for 5 minutes to exchange the ions adsorbed on the roots. Then, the roots and shoots were harvested. Following 30 minutes of 105 °C and 3 d of 80 °C drying, the dry weight of the plants was determined by a balance. The dry sample was powdered using a ball mill and then passed through a 0.425 mm sieve. Each sample containing 0.05 g of the uniform powder was set into a carbon and nitrogen element analyser for the carbon and nitrogen contents determination. Total root carbon content = root carbon content × root dry weight. Total shoot carbon content = shoot carbon content × shoot dry weight. Whole plant carbon content = total root carbon content + total shoot carbon content. Similarly, the total root nitrogen content, total shoot nitrogen content, and whole plant nitrogen content are obtained.

### 2.6 Ammonium and nitrate uptake rates

The method used for determining ammonium and nitrate uptake rates was based on our previous reports (Hao et al., 2020). Briefly, centipedegrass plants were subjected to nitrogen starvation (by cultivation in a nutrient solution without nitrogen) for 3 days followed by one month of different NH_4_^+^:NO_3_^-^ ratio treatments. Then, the roots were soaked in a 0.1 mM CaSO_4_ solution for 5 minutes to exchange the ions sticking to the root. After cleaning with water, the roots were placed into an ammonium uptake solution containing 0.1 mM NH_4_Cl and 0.1 mM CaSO_4_ (pH 5.5) or a nitrate uptake solution containing 0.1 mM NaNO_3_ and 0.1 mM CaSO_4_ (pH 5.5) for 1 hour. Each seedling was carefully placed in a 250 mL container, and its roots were immersed in either 200 mL of ammonium uptake solution or nitrate uptake solution. The residual fluid was then collected, and the fresh weight of the corresponding roots was simultaneously measured. The concentrations of ammonium and nitrate retained in the uptake solution were determined by Nessler’s reagent method (for ammonium, #S26016) and the sulfonamide colorimetric method (for nitrate, #R30301-100T) according to the manufacturer’s instructions (Shanghai Yuanye Biological Technology Company, China), respectively. The nitrogen absorption rate was determined with the following equation: nitrogen absorption rate = (initial concentration – sample concentration) × volume/(absorption time × root weight).

### 2.7 Glutamine, fructose, glucose, sucrose, and starch content determinations

Glutamine (Gln) content was determined by the high-performance liquid chromatography (HPLC) method (Pilot et al., 2004). The contents of fructose, glucose, sucrose, and starch were determined according to the instructions of the Nanjing Jiancheng Company reagent kit (#MB-W-B501 for fructose, #MB-W-B500 for glucose, #MB-W-B502 for sucrose, #MB-W-C400 for starch). A colorimetric method was used. The determination of fructose, glucose, sucrose, and starch content was performed at wavelengths of 480 nm, 505 nm, 480 nm, and 620 nm, respectively.

### 2.8 Statistical analysis and graph drawing

All the data were analysed using one-way analysis of variance (ANOVA) followed by Duncan’s *post hoc* multiple comparisons tests (P < 0.05). Figures were drawn by GraphPad Prism 9.5. Data are expressed as the mean ± standard error (SE) of at least three measurements.

## 3 Results

### 3.1 Plant growth

**Figure 1:**
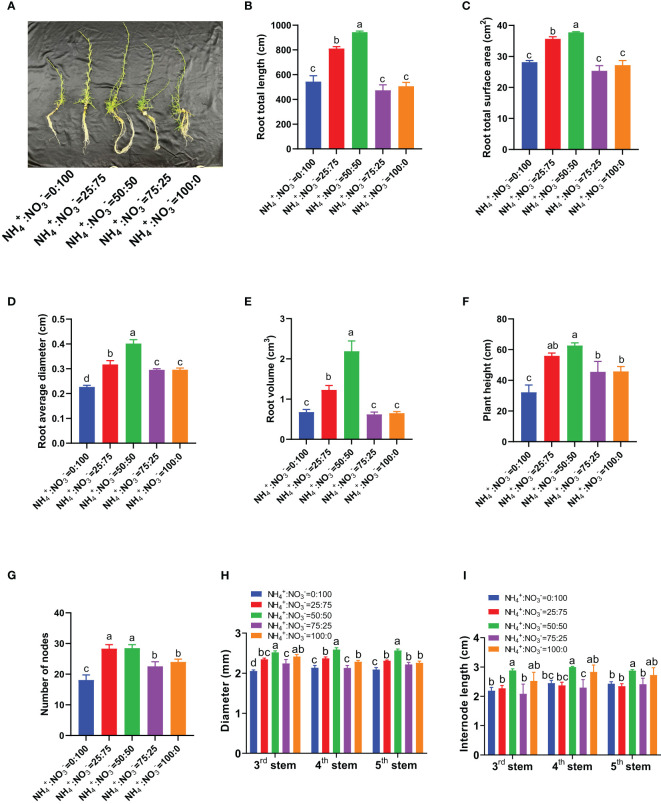
Growth parameters of centipedegrass that received one month of different NH_4_^+^:NO_3_^-^ ratio treatments. **(A)**, Photos of growth. **(B)**, Root total length. **(C)**, Root total surface area. **(D)**, Root average diameter. **(E)**, Root volume. **(F)**, Plant height. **(G)**, Numbers of nodes. **(H)**, Diameter of the 3^rd^, 4^th^, and 5^th^ stems. **(I)**, Internode length of the 3^rd^, 4^th^, and 5^th^ stems. N=4 for Panels **(B–E)**, n=8 for Panels **(F–I)**. The different letters above the columns represent significant differences between treatments (P < 0.05).

Under hydroponic conditions, a of five NH_4_^+^:NO_3_^-^ ratios (0:100; 25:75; 50:50; 75:25; 100:0) with a constant total nitrogen concentration was set to study the effect of changing the NH_4_^+^:NO_3_^-^ ratio on the growth of centipedegrass. The results showed that as the proportion of ammonium increased, the growth response showed a “bell-shaped” pattern (first increasing and then decreasing), achieving the maximum growth effect at an NH_4_^+^:NO_3_^-^ ratio of 50:50 (**Figure 1A**). With the increase in the ammonium proportion, the total root length, total root surface area, average diameter, and root volume all showed a trend of first increasing and then decreasing. The maximum root growth parameters were obtained at an NH_4_^+^:NO_3_^-^ ratio of 50:50 (**Figures 1B-E**). Compared with the situation under the supply of only nitrate nitrogen, the total root length increased by 49.2%, 73.6%, -12.8%, and -6.7% (**Figure 1B**), the total root surface area increased by 26.6%, 34.0%, -9.9%, and -3.4% (**Figure 1C**), the average root diameter increased by 39.9%, 77.1%, 30.4%, and 30.6% (**Figure 1D**), and the root volume increased by 81.1%, 223.3%, -8.4%, and -4.2% (**Figure 1E**) with the increase in the proportion of ammonium. The plant height and number of stem nodes also showed a trend of first increasing and then decreasing with an increasing proportion of ammonium, reaching their maximum values at NH_4_^+^:NO_3_^-^ ratios of 50:50 and 25:75 (**Figures 1F, G**). Compared with the situation where only nitrate was supplied, the plant height increased by 73.7%, 94.5%, 41.4%, and 42.1% after the proportion of ammonium increased (**Figure 1F**); the number of stem nodes increased by 56.3%, 57.2%, 24.5%, and 32.4% (**Figure 1G**). As the proportion of ammonium increased, the diameter and internode length of the 3^rd^, 4^th^, and 5^th^ stem nodes showed a trend of first increasing and then decreasing. The NH_4_^+^:NO_3_^-^ ratio of 50:50 treatment had the largest stem diameter and internode length (**Figures 1H, I**). Compared with the situation where only nitrate was supplied, the diameter of the 3^rd^ stem nodes increased by 14.1%, 22.7%, 9.2%, and 17.5%, the diameter of the 4^th^ stem nodes increased by 10.9%, 21.2%, -0.2%, and 7.0%, and the diameter of the 5^th^ stem nodes increased by 10.4%, 22.7%, 6.1%, and 8.0% (**Figure 1H**). The internode length of the 3^rd^ stem nodes increased by 3.58%, 31.5%, -4.9%, and 15.1%, the internode length of the 4^th^ stem nodes increased by -3.2%, 22.4%, -6.1%, and 15.6%, and the internode length of the 5^th^ stem nodes increased by -3.6%, 18.4%, -0.7%, and 12.1% (**Figure 1I**).

### 3.2 Micromorphological structure

**Figure 2:**
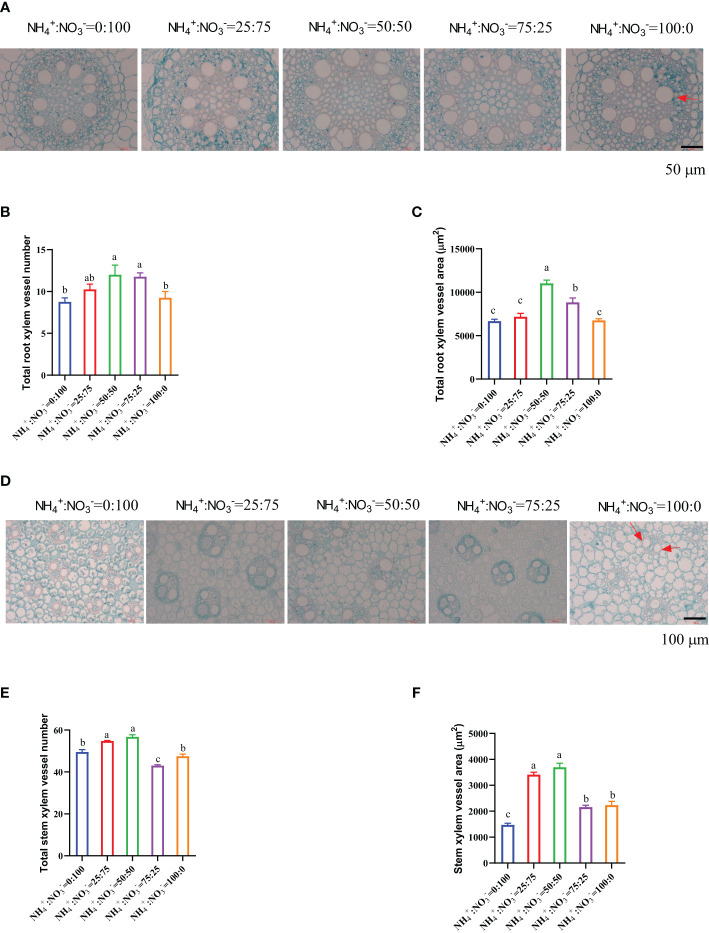
Cross-sections of the roots and stems that received one month of different NH_4_^+^:NO_3_^-^ ratio treatments. **(A–C)**, Cross section of root mature zone **(A)**, total xylem vessel number **(B)**, and total xylem vessel **(C)**. **(D–F)**, Cross section of the 5^th^ stem **(D)**, total stem xylem vessel number **(E)**, and stem xylem vessel area **(F)**. N=4 for Panels **B**, **C**, and **E**, n=16 for Panel **(F)** The red arrows in **(A, D)** indicate the root vessel and stem vessel, respectively. The different letters above the columns represent significant differences between treatments (P < 0.05).

Because the growth of the root and stem was regulated by the treatment of different NH_4_^+^:NO_3_^-^ ratios (**Figure 1D**; **Figure 1H**), the root and stem were cross-cut to observe their micromorphological structure changes. The results showed that the number and area of xylem vessels in the root mature zone showed a trend of first increasing and then decreasing with increasing ammonium proportion. The NH_4_^+^:NO_3_^-^ ratio of 50:50 treatment had the largest number and area of root xylem vessels (**Figures 2A-C**). Compared with only nitrate supplied, increasing the proportion of ammonium increased the number of root xylem vessels by 17.1%, 37.1%, 34.3%, and 5.7%, respectively (**Figure 2B**), and increased the xylem vessel area by 7.7%, 65.2%, 32.3%, and 1.3%, respectively (**Figure 2C**). The number and area of xylem vessels in the stem also showed a trend of first increasing and then decreasing with increasing ammonium proportion. The NH_4_^+^:NO_3_^-^ ratio of 50:50 and 25:75 treatments had the largest number and area of stem xylem vessels (**Figures 2D-F**). Compared with the only nitrate supplied, increasing the proportion of ammonium increased the number of stem xylem vessels by 10.6%, 14.6%, -13.1%, and -4.0% (**Figure 2E**) and increased the stem xylem vessel area by 131.5%, 150.9%, 46.3%, and 51.7% (**Figure 2F**).

### 3.3 Biomass, carbon, and nitrogen content

**Figure 3:**
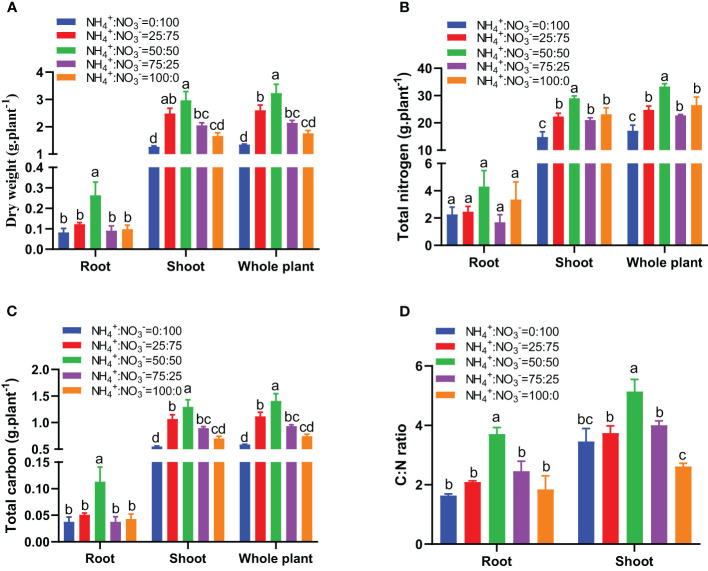
The biomass, carbon, and nitrogen accumulation in centipedegrass that received one month of different NH_4_^+^:NO_3_^-^ ratio treatments. **(A)**, Biomass. **(B)**, Nitrogen accumulation. **(C)**, Carbon accumulation. **(D)**, Carbon **(C)** nitrogen (N) ratio. N=4. The different letters above the columns represent significant differences between treatments (P < 0.05).

With the increase in the ammonium proportion, the biomass showed a trend of first increasing and then decreasing. The NH_4_^+^:NO_3_^-^ ratio of 50:50 treatment had the maximum biomass of roots, shoots, and whole plants (**Figure 3A**). Compared with the situation where nitrate was supplied alone, increasing the ammonium proportion increased the root biomass by 48.6%, 218.4%, 9.9%, and 19.0%, the shoot biomass by 96.1%, 134.6%, 62.2%, and 31.2%, and the whole plant biomass by 93.2%, 139.7%, 59.0%, and 30.5% (**Figure 3A**). The nitrogen accumulation, carbon accumulation, and carbon-nitrogen ratio also showed a trend of first increasing and then decreasing with increasing ammonium proportion. The NH_4_^+^:NO_3_^-^ ratio of 50:50 treatment showed the highest nitrogen accumulation, carbon accumulation, and carbon-nitrogen ratio (**Figures 3B-D**). Compared with only nitrate being supplied, increasing the ammonium proportion increased the root nitrogen accumulation by 9.0%, 90.1%, -25.5%, and 48.1%, the shoot nitrogen accumulation by 50.4%, 95.3%, 41.8%, and 55.9%, and the whole plant nitrogen accumulation by 44.9%, 94.6%, 32.8%, and 54.8% (**Figure 3B**). Compared with the situation where only nitrate was supplied, increasing the ammonium proportion increased the root carbon accumulation by 34.8%, 200.2%, 0.1%, and 13.8%, the shoot carbon accumulation by 94.2%, 135.8%, 62.8%, and 27.5%, and the whole plant carbon accumulation by 90.4%, 139.9%, 58.7%, and 26.6% (**Figure 3C**). Compared with the situation where nitrate was supplied alone, increasing the ammonium proportion increased the root carbon:nitrogen ratio by 27.7%, 126.1%, 49.9%, and 12.4% and the shoot carbon:nitrogen ratio by 8.3%, 48.7%, 15.9%, and -24.3% (**Figure 3D**).

### 3.4 Nitrogen uptake and assimilation

**Figure 4:**
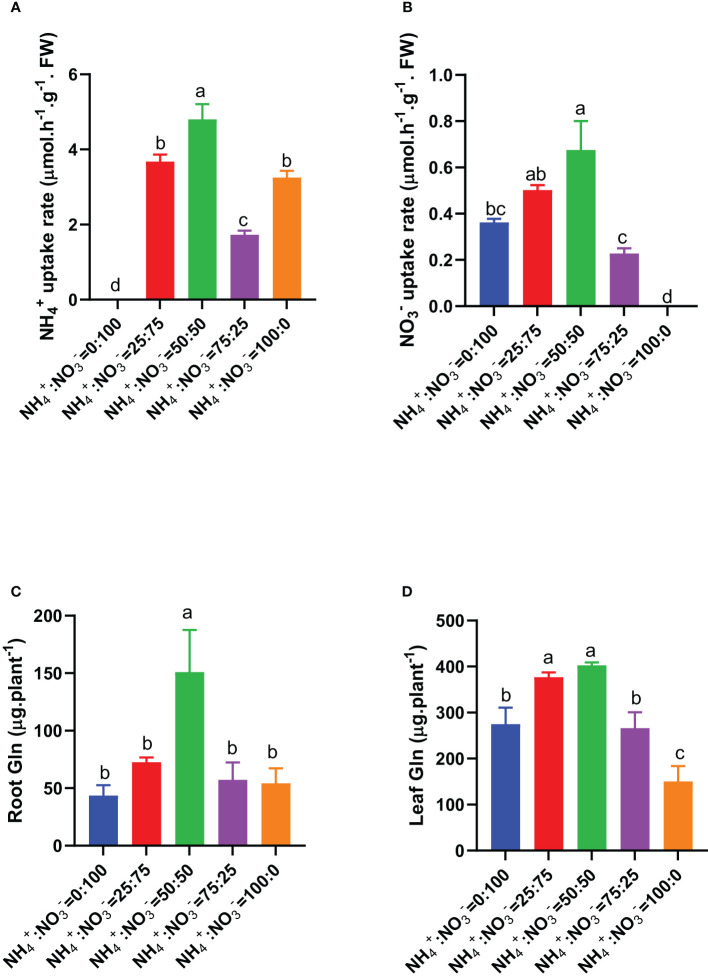
Ammonium and nitrate uptake rates in centipedegrass that received one month of different NH_4_^+^:NO_3_^-^ ratio treatments. **(A)**, Ammonium uptake rate. **(B)**, Nitrate uptake rate. **(C, D)**, Total Gln in the roots and leaves. N=4. The different letters above the columns represent significant differences between treatments (P < 0.05).

Since roots are the main pathway of nitrogen input, we first measured the absorption rate of ammonium nitrate by roots that received different NH_4_^+^:NO_3_^-^ ratio treatments. The results showed that as the proportion of ammonium increased, the absorption rates of ammonium and nitrate showed a trend of first increasing and then decreasing. The maximum absorption rates of ammonium and nitrate were observed under the NH_4_^+^:NO_3_^-^ ratio of 50:50 treatment (**Figures 4A, B**). No matter NH_4_^+^ or NO_3_^-^, they need to be first assimilated into Gln through the GS-GOGAT pathway. The first nitrogen assimilation product Gln in roots and leaves also showed a trend of first increasing and then decreasing with increasing ammonium proportion. The highest Gln content was observed under the NH_4_^+^:NO_3_^-^ ratio of 50:50 treatment (**Figures 4C, D**). Compared with the situation where nitrate was supplied alone, increasing the ammonium proportion increased the root total Gln content by 66.1%, 245.9%, 31.0%, and 24.2% (**Figure 4C**) and the shoot total Gln content by 37.2%, 46.5%, -3.2%, and -45.3% (**Figure 4D**).

### 3.5 Carbon uptake and assimilation

**Figure 5:**
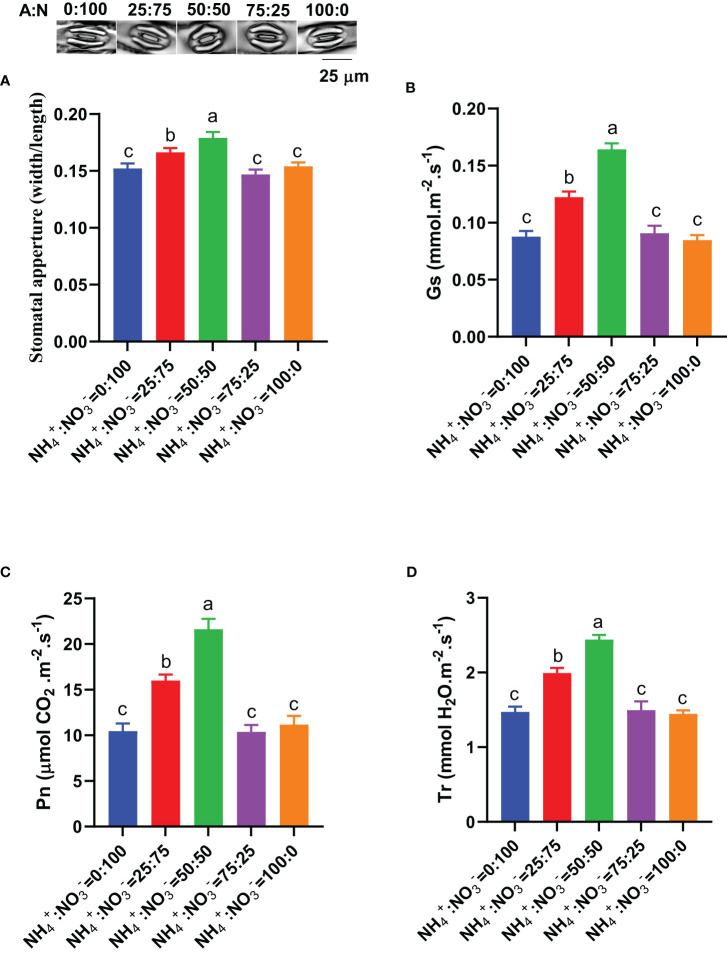
Stomatal aperture and photosynthetic parameters in centipedegrass that received one month of different NH_4_^+^:NO_3_^-^ ratio treatments. **(A)**, Representative photos of stomata (upper) and statistical results of stomatal aperture (lower). **(B)**, Stomatal conductance (Gs). **(C)**, Photosynthetic rate (Pn). **(D)**, Transpiration rate (Tr). N>80 for Panel **(A)**. n=4 for Panels **(B–D)**. The different letters above the columns represent significant differences between treatments (P < 0.05).

The stomata located on the leaves are the gateway for plant carbon input. We monitored the stomatal aperture under different NH_4_^+^:NO_3_^-^ ratio treatments and found that the stomatal aperture showed a trend of first increasing and then decreasing with increasing proportions of ammonium, reaching the maximum stomatal opening at an NH_4_^+^:NO_3_^-^ ratio of 50:50 (**Figure 5A**). The trend of stomatal conductance measurement data is consistent with that of stomatal aperture measurement data, reaching the maximum stomatal conductance at an NH_4_^+^:NO_3_^-^ ratio of 50:50 (**Figure 5B**). Compared with the situation where only nitrate was supplied, increasing the ammonium proportion increased the stomatal conductance by 39.6%, 87.2%, 3.4%, and -3.4% (**Figure 5B**). Consistent with the trend of changes in stomatal aperture/conductance, both the photosynthetic rate and transpiration rate showed a trend of first increasing and then decreasing with increasing ammonium proportion, reaching the maximum photosynthetic rate and transpiration rate at an NH_4_^+^:NO_3_^-^ ratio of 50:50 (**Figures 5C, D**). Compared with the situation where nitrate was supplied alone, increasing the ammonium proportion increased the photosynthetic rate by 52.7%, 106.3%, -1.0%, and 6.8% (**Figure 5C**) and the transpiration rate by 35.3%, 65.6%, 1.5%, and -1.9% (**Figure 5D**).

**Figure 6:**
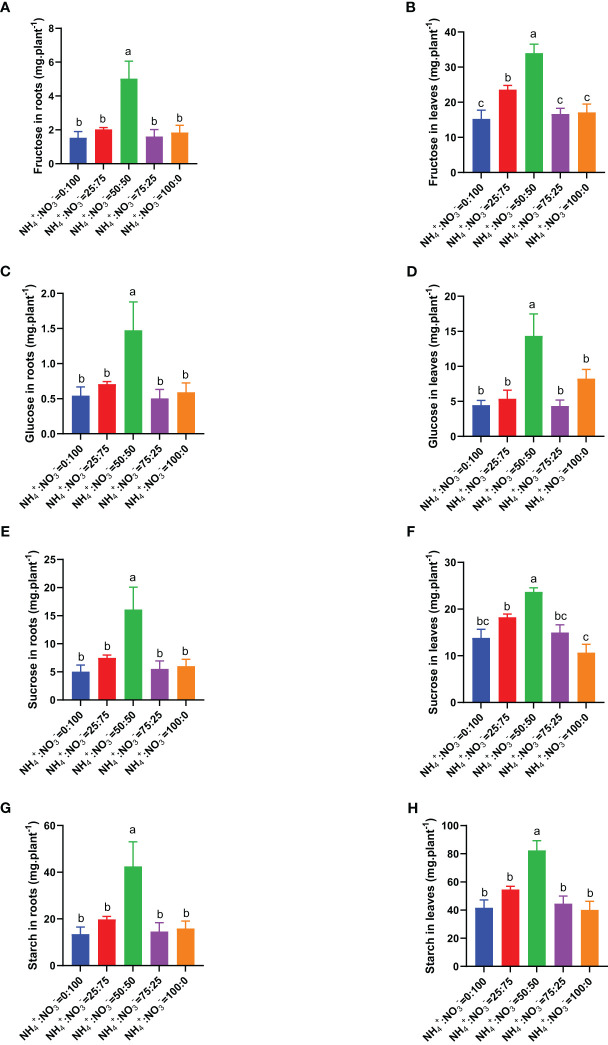
Total soluble sugars and starch in centipedegrass that received one month of different NH_4_^+^:NO_3_^-^ ratio treatments. **(A, B)**, Total fructose content in the roots **(A)** and leaves **(B)**. **(C, D)**, Total glucose content in the roots **(C)** and leaves **(D)**. **(E, F)**, Total sucrose content in the roots **(E)** and leaves **(F)**. **(G, H)**, Total sucrose content in the roots **(G)** and leaves **(H)**. N=4. The different letters above the columns represent significant differences between treatments (P < 0.05).

Considering that soluble sugar and starch are the main photosynthetic products, we measured these parameters under different NH_4_^+^:NO_3_^-^ ratio treatments. The results showed that the contents of fructose, glucose, sucrose, and starch in roots and leaves showed a trend of first increasing and then decreasing with increasing ammonium proportion. NH_4_^+^:NO_3_^-^ ratio of 50:50 had the highest content of fructose, glucose, sucrose, and starch (**Figures 6A-H**). Compared with the situation where only nitrate was supplied, increasing the ammonium proportion increased the content of fructose in roots by 32.3%, 227.9%, 4.9%, and 20.1% (**Figure 6A**), the content of fructose in leaves by 54.4%, 122.2%, 8.8%, and 11.9% (**Figure 6B**), the content of glucose in roots by 30.3%, 171.1%, -7.3%, and 8.6% (**Figure 6C**), the content of glucose in leaves by 20.6%, 221.4%, -2.7%, and 84.8% (**Figure 6D**), the content of sucrose in roots by 48.3%, 218.5%, 9.3%, and 19.5% (**Figure 6E**), the content of sucrose in leaves by 31.8%, 71.3%, 8.3%, and -22.8% (**Figure 6F**), the content of starch in roots by 46.9%, 215.1%, 8.5%, and 17.8% (**Figure 6G**), and the content of starch in leaves by 31.0%, 97.9%, 7.2%, and -3.7% (**Figure 6H**).

### 3.6 Early stage response of stomata

**Figure 7:**
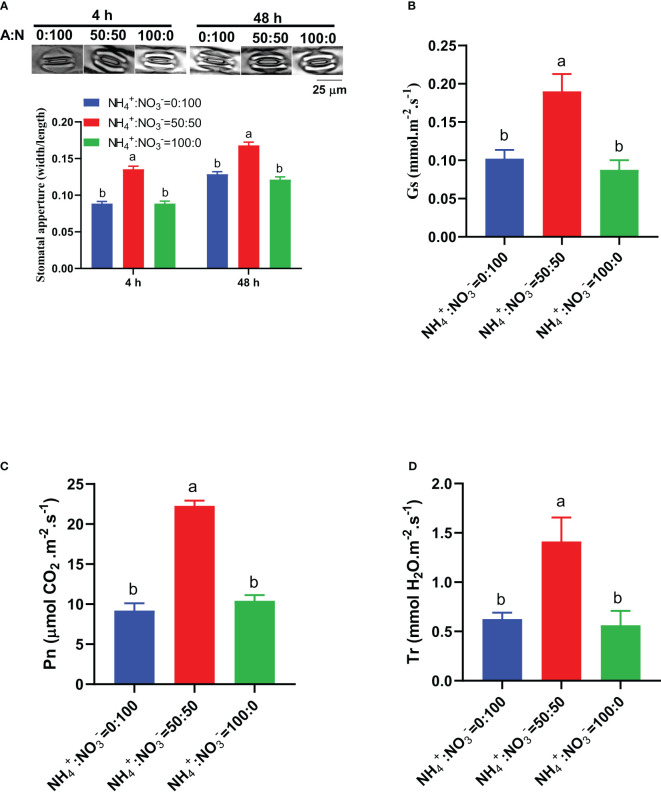
The early stage response of stomata and photosynthetic parameters to different NH_4_^+^:NO_3_^-^ ratio treatments. **(A)**, Representative photos of stomata (upper) and statistical results of stomatal aperture (lower) in leaves that received 4 h and 48 h of treatments. **(B–D)**, Stomatal conductance (Gs) **(B)**, photosynthetic rate (Pn) **(C)**, and transpiration rate (Tr) **(D)** in leaves that received 48 hours of treatments. N>70 for Panel **(A)**. n=4 for Panels **(B–D)**. The different letters above the columns represent significant differences between treatments (P < 0.05).

In view of the key roles of stomata and photosynthesis in carbon input and assimilation, we monitored the early-stage response of these parameters to different NH_4_^+^:NO_3_^-^ ratio treatments. The results showed that 4 hours after being treated with different NH_4_^+^:NO_3_^-^ ratios, the NH_4_^+^:NO_3_^-^ ratio of 50:50 treatment had the largest stomatal aperture, and this situation was maintained with the extension of treatment time (48 hours) (**Figure 7A**). At the two time points of 4 hours and 48 hours, the stomatal aperture under the NH_4_^+^:NO_3_^-^ ratio of 50:50 treatment increased by 52.8% and 30.6% and by 52.8% and 38.5%, respectively, compared to those under only nitrate or only ammonium conditions (**Figure 7A**). The stomatal conductance also showed similar response characteristics: the maximum stomatal conductance was reached at the NH_4_^+^:NO_3_^-^ ratio of 50:50 treatment, which increased by 85.7% and 117.1%, respectively, compared to those under only nitrate and only ammonium treatments (**Figure 7B**). The photosynthetic rate reached its maximum under the NH_4_^+^:NO_3_^-^ ratio of 50:50 treatment, which increased by 142.0% and 113.6% compared to those under only nitrate or only ammonium conditions, respectively (**Figure 7C**). The transpiration rate reached its maximum under the NH_4_^+^:NO_3_^-^ ratio of 50:50 treatment, which increased by 126.1% and 151.2% compared to those under only nitrate or only ammonium conditions, respectively (**Figure 7D**).

## 4 Discussion

### 4.1 Appropriate NH_4_^+^:NO_3_^-^ ratio promotes the growth and improves the carbon sequestration ability and nitrogen use efficiency of centipedegrass

The appropriate NH_4_^+^:NO_3_^-^ ratio promotes plant growth, and different species require different optimal NH_4_^+^:NO_3_^-^ ratios. For example, the optimal NH_4_^+^:NO_3_^-^ ratio is 0:100 for yellow flag (Chang et al., 2010) and wheat (Yang et al., 2021); 25:75 for Chinese cabbage cultivars (Chen et al., 2005; Zhu et al., 2021), strawberry (Tabatabaei et al., 2006), tomato (Liu et al., 2017), Chinese kale (Wang et al., 2021), and lettuce (Du et al., 2022); 50:50 for cabbage (Zhang et al., 2007), Sweet flag (Chang et al., 2010), coffee (Carr et al., 2020), soybean (Raza et al., 2021), and blackberry (Wei et al., 2023); 75:25 for pecan (Chen et al., 2021); and 83:17 for blueberry (Zhang et al., 2021). In this study, the effects of different NH_4_^+^:NO_3_^-^ ratios on the growth parameters of centipedegrass were monitored and the NH_4_^+^:NO_3_^-^ ratio of 50:50 treatment had the highest biomass and carbon and nitrogen accumulation (**Figure 3**). We recommend an NH_4_^+^:NO_3_^-^ ratio of 50:50 as the optimal NH_4_^+^:NO_3_^-^ ratio for the production of centipedegrass. The reason for this phenomenon is as follows. 1) Roots, the gateway for nitrogen input, have the maximal ammonium and nitrate absorption capacity, which is closely related to the better root architecture under this treatment (**Figure 1**; **Figure 4**). Certainly, the possibility that the increased expression abundance of transporters responsible for ammonium and nitrate uptake contributes to the maximal nitrogen uptake capacity cannot be ruled out. In addition to the improvement of nitrogen absorption capacity, the NH_4_^+^:NO_3_^-^ ratio of 50:50 has the strongest nitrogen assimilation ability, which is confirmed by the highest Gln (the first product of nitrogen assimilation) accumulation (**Figure 4**). As an essential element, the improvement of both the absorption and utilization capacity of nitrogen is beneficial for plant growth. The enhanced nitrogen assimilation ability requires more energy and carbon skeleton, which were come from the enhanced carbon absorption and assimilation ability mentioned below. 2) Leaves, the gateway for carbon input, have the highest photosynthetic capacity and content of carbohydrates (fructose, glucose, sucrose, starch), which is closely related to the largest stomatal aperture under this treatment (**Figures 5**, **6**). The photosynthetic rate reflects the ability of plants to assimilate CO_2_. The higher the photosynthetic rate demonstrates a stronger ability of plants to assimilate CO_2_. The photosynthetic products of CO_2_ assimilation are sugars, which are composed of monosaccharides (glucose, fructose), disaccharides (sucrose), and polysaccharides (starch). Therefore, the NH_4_^+^:NO_3_^-^ ratio of 50:50 resulted in the highest photosynthetic rate, resulting in the maximum photosynthetic products. This is similar to the result of the maximum sugar accumulation in corn under the optimal NH_4_^+^:NO_3_^-^ ratio (Wang et al., 2019b). Since more than 90% of the dry matter accumulation in plants comes from photosynthesis, the increase in photosynthetic rate and products is beneficial for plant growth. 3) The NH_4_^+^:NO_3_^-^ ratio of 50:50 treatment showed the largest stem xylem vessel area and transpiration rate (**Figure 2**; **Figure 5**). The NH_4_^+^:NO_3_^-^ ratio of 50:50 treatment had the highest transpiration rate, which provides plants with greater transpiration pull and facilitates the root-to-shoot translocation of water and inorganic salts from the enlarged stem xylem vessel. Among the three explanations, the stronger photosynthetic capacity and better root architecture are consistent with many reports of appropriate NH_4_^+^:NO_3_^-^ ratios promoting plant growth (Tabatabaei et al., 2006; Hu et al., 2017; Liu et al., 2017; Wang et al., 2019b; Carr et al., 2020; Raza et al., 2021; Zhang et al., 2021). There is still a lack of information regarding the response of the micromorphological structure of roots and stems to changes in the NH_4_^+^:NO_3_^-^ ratio. This study was the first to report the effect of different NH_4_^+^:NO_3_^-^ ratios on the micromorphological structure of roots and stems. The NH_4_^+^:NO_3_^-^ ratio of 50:50 treatment had the highest number of root xylem vessels, root xylem vessel area, stem xylem vessel number, and stem xylem vessel area (**Figure 2**). Given that a larger root and stem xylem vessel area is conducive to the loading of water and inorganic salts in the roots and their root-to-shoot translocation (Albornoz et al., 2020) and that the NH_4_^+^:NO_3_^-^ ratio of 50:50 treatment had the highest transpiration rate, the greater transpiration pull and the enlarged stem xylem vessel facilitated nutrient uptake and translocation.

The mean biomass carbon density of grasslands (4.8 Mg C ha^−1^) in China is significantly lower than the global average level (7.2 Mg C ·ha^−1^) (Tang et al., 2018), indicating that grasslands in China still have high carbon sequestration potential. In this study, the maximum carbon accumulation was achieved under the NH_4_^+^:NO_3_^-^ ratio of 50:50 treatment, which significantly improved the carbon sequestration capacity, reaching an increased amplitude of 140% when compared with the parameter under only nitrate treatment (**Figure 3**). This appropriate NH_4_^+^:NO_3_^-^ ratio is efficient in enhancing the carbon sequestration capacity of grasslands. The reason for the maximum carbon accumulation achieved under the NH_4_^+^:NO_3_^-^ ratio of 50:50 treatment is attributed to the maximum stomatal aperture, stomatal conductivity, maximum photosynthetic rate, and maximum soluble sugar and starch content (**Figures 5**, **6**). Numerous reports have shown that an appropriate NH_4_^+^:NO_3_^-^ ratio improves the photosynthetic rate (Tabatabaei et al., 2006; Hu et al., 2017; Liu et al., 2017; Wang et al., 2019b; Carr et al., 2020; Raza et al., 2021; Zhang et al., 2021). The underlying mechanism is related to the maximum and effective quantum yield of PSII, improved activities of Calvin cycle enzymes, increased levels of mRNA relative expression of several genes involved in the Calvin cycle (Hu et al., 2017; Raza et al., 2021), improved gas exchanges (Raza et al., 2021), and higher chlorophyll pigments. The positive correlation between photosynthetic rate and stomatal aperture/conductance observed in this study further confirms the above conclusion (**Figure 5**). The increase in stomatal opening occurred in the early stages of treatment (4 hours) (**Figure 7**), indicating that the change in stomatal opening is crucial for the improved photosynthetic rate. Whether other factors affecting the photosynthetic rate changed in the early stages of NH_4_^+^:NO_3_^-^ ratio treatment, thereby regulating the photosynthetic rate, needs further experimental evidence.

The improvement of nitrogen use efficiency can effectively reduce a series of environmental problems caused by large nitrogen fertilizer production and application. The maximum nitrogen accumulation was achieved under the NH_4_^+^:NO_3_^-^ ratio of 50:50 treatment, which significantly improved the nitrogen use efficiency compared to other NH_4_^+^:NO_3_^-^ ratio treatments (**Figure 3**). We recommend an NH_4_^+^:NO_3_^-^ ratio of 50:50 as the optimal NH_4_^+^:NO_3_^-^ ratio to improve nitrogen use efficiency. The highest nitrogen accumulation was attributed to the highest root ammonium, nitrate uptake rate, and assimilation ability (**Figure 4**). The NH_4_^+^:NO_3_^-^ ratio of 50:50 treatment resulted in the largest root surface area and total root length (**Figure 1**). The root architecture plays an essential role in nitrogen uptake (Meister et al., 2014). The root length and surface area are important for nitrogen acquisition and a large root system is associated with higher nitrogen acquisition (Sattelmacher et al., 1990), since these parameters reflect the contact area between the plant root system and the external nutrient solution. The larger the total root length and root surface area would lead to a greater contact area between the plant and the rhizosphere nutrient, which is more conducive to nutrient absorption under the NH_4_^+^:NO_3_^-^ ratio of 50:50 treatment. In addition, an increase in the area of the root xylem vessel is also beneficial for nutrient loading in the roots (**Figure 2**). The absorption rate of ammonium was much higher than that of nitrate (**Figure 4**), indicating that centipedegrass has a preference for ammonium. This may reflect an adaptation of centipedegrass to its acidic soil growth environment, similar to the situation in other plants (Daryanto et al., 2019). The NH_4_^+^:NO_3_^-^ ratio of 50:50 treatment had the highest Gln content, which may be closely related to the maximum photosynthetic rate and the largest amount of photosynthetic products, providing the carbon skeleton and energy needed for nitrogen assimilation (Li et al., 2020).

### 4.2 Stomata are the structures that respond to root ammonium nitrate ratio treatment at the early stage

Carbon-nitrogen synergy is beneficial for plant growth (Wang et al., 2019a). Carbon metabolism provides energy and carbon skeletons for nitrogen metabolism. Nitrogen, as a component of amino acids, proteins, nucleic acids, phospholipids, enzymes, ATP, chlorophyll, and hormones, significantly regulates carbon metabolism (Lawlor, 2002). The main reason for plant growth inhibition under ammonium supply alone is photosynthetic inhibition-induced carbon deficiency (Yang et al., 2020). The discovery in this study that ammonium treatment alone had the smallest carbon accumulation and carbon-nitrogen ratio once again supports this conclusion (**Figure 3**). Compared with other NH_4_^+^:NO_3_^-^ ratio treatments, the NH_4_^+^:NO_3_^-^ ratio of 50:50 treatment showed the highest stomatal opening and photosynthetic rate, resulting in the highest carbon accumulation and carbon-nitrogen ratio (**Figure 3**). Considering that photosynthesis is the main source of plant carbon, the maximum photosynthetic rate under the NH_4_^+^:NO_3_^-^ ratio of 50:50 treatment is the main reason for its maximum carbon accumulation and carbon-nitrogen ratio under this treatment. Numerous studies have reported the key role of enhanced photosynthetic capacity in promoting plant growth under appropriate NH_4_^+^:NO_3_^-^ ratio treatments. Notably, those measurements were obtained after long-term NH_4_^+^:NO_3_^-^ ratio treatment (>12 days) (Tabatabaei et al., 2006; Wang et al., 2019b; Zhang et al., 2021). This study was the first to show that in the early stages of treatment (4 hours), stomata began to respond to the root NH_4_^+^:NO_3_^-^ ratio supply (**Figure 7**). Manipulating stomata is a promising focus for improving the nitrogen use efficiency of centipedegrass.

## 5 Conclusion

**Figure 8:**
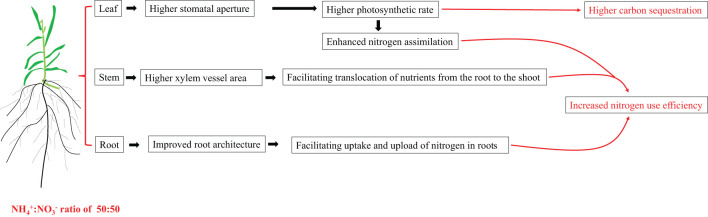
A mode explaining the growth promotion caused by the appropriate NH_4_^+^:NO_3_^-^ ratio treatment. Under the appropriate NH_4_^+^:NO_3_^-^ ratio treatment, the changes in root architecture allow a more favourable absorption of nitrogen and its loading into the root xylem. The greater transpiration pull triggered by greater transpiration and the larger stem xylem vessel synergistically facilitated the root-to-shoot translocation of nutrients. The increase in leaf stomatal opening increases the photosynthetic rate, increases carbon absorption and assimilation, and promotes nitrogen assimilation. The synergistic changes in the structure and physiological functions of the three organs of roots, stems, and leaves caused by the appropriate NH_4_^+^:NO_3_^-^ ratio together improve the nitrogen use efficiency and carbon sequestration capacity of centipedegra.

We recommend 50:50 as the appropriate NH_4_^+^:NO_3_^-^ ratio for the growth of centipedegrass, which not only improves the nitrogen use efficiency but also enhances the carbon sequestration capacity. Both of which facilitate the achievement of carbon neutrality. The synergistic effect of physiological and structural aspects on various parts of roots, stems, and leaves is the main reason for this phenomenon. Briefly, the changes in root architecture allow a more favourable absorption of nitrogen and its loading into the root xylem. The greater transpiration pull triggered by greater transpiration and the larger stem xylem vessel synergistically facilitated the root-to-shoot translocation of nutrients. The increase in leaf stomatal opening increases the photosynthetic rate, increases carbon absorption and assimilation, and promotes nitrogen assimilation. The synergistic changes in the structure and physiological functions of the three organs of roots, stems, and leaves caused by the appropriate NH_4_^+^:NO_3_^-^ ratio together improve the nitrogen use efficiency and carbon sequestration capacity of centipedegrass (**Figure 8**). Considering that stomatal aperture is sensitive to the root NH_4_^+^:NO_3_^-^ ratio treatment, manipulating stomata is a promising strategy for improving the nitrogen use efficiency of centipedegrass.

## Data availability statement

The original contributions presented in the study are included in the article/supplementary material. Further inquiries can be directed to the corresponding authors.

## Author contributions

DH: Data curation, Methodology, Writing – original draft. JZ: Methodology, Writing – review & editing. LL: Writing – review & editing. JQ: Methodology, Writing – review & editing. XL: Writing – review & editing. RC: Writing – review & editing. WK: Writing – review & editing. DL: Writing – review & editing. JL: Writing – review & editing. HG: Writing – review & editing. JL: Writing – review & editing. JZ: Supervision, Writing – review & editing. JC: Supervision, Writing – review & editing.
